# A review of models applied to the geographic spread of Zika virus

**DOI:** 10.1093/trstmh/trab009

**Published:** 2021-02-11

**Authors:** Sabrina L Li, Jane P Messina, Oliver G Pybus, Moritz U G Kraemer, Lauren Gardner

**Affiliations:** School of Geography and the Environment, University of Oxford, South Parks Road, Oxford, OX1 3QY, UK; School of Geography and the Environment, University of Oxford, South Parks Road, Oxford, OX1 3QY, UK; School of Global and Area Studies, University of Oxford, 12 Bevington Road, Oxford, OX2 6LH, UK; Department of Zoology, University of Oxford, 11a Mansfield Rd, Oxford, OX1 3SZ, UK; Department of Zoology, University of Oxford, 11a Mansfield Rd, Oxford, OX1 3SZ, UK; Department of Civil and Systems Engineering, Johns Hopkins University, 3400 North Charles Street, Baltimore, MD, 21218-2682, USA; Center for Systems Science and Engineering, Johns Hopkins University, 3400 North Charles Street, Baltimore, MD, 21218-2682, USA

**Keywords:** arbovirus, mobility, surveillance, vector-borne, ZIKV

## Abstract

In recent years, Zika virus (ZIKV) has expanded its geographic range and in 2015–2016 caused a substantial epidemic linked to a surge in developmental and neurological complications in newborns. Mathematical models are powerful tools for assessing ZIKV spread and can reveal important information for preventing future outbreaks. We reviewed the literature and retrieved modelling studies that were developed to understand the spatial epidemiology of ZIKV spread and risk. We classified studies by type, scale, aim and applications and discussed their characteristics, strengths and limitations. We examined the main objectives of these models and evaluated the effectiveness of integrating epidemiological and phylogeographic data, along with socioenvironmental risk factors that are known to contribute to vector–human transmission. We also assessed the promising application of human mobility data as a real-time indicator of ZIKV spread. Lastly, we summarised model validation methods used in studies to ensure accuracy in models and modelled outcomes. Models are helpful for understanding ZIKV spread and their characteristics should be carefully considered when developing future modelling studies to improve arbovirus surveillance.

## Introduction

Zika virus (ZIKV) is transmitted to humans via the bite of infected *Aedes* sp. mosquitoes. Since the virus was first isolated in 1947, ZIKV has circulated for decades without causing large reported outbreaks or severe disease.^1^ The first known outbreak occurred on Yap Island in the Western Pacific in 2007, but no hospitalisations or deaths were reported.^2^ ZIKV then reached French Polynesia and the South Pacific in 2013 and was introduced to Brazil in late 2015, likely through international air travel.^3^ The Zika epidemic in Brazil received global attention when a strong epidemiological link was established with an increase in cases of microcephaly.^4^ Subsequently ZIKV was declared a Public Health Emergency of International Concern by the WHO^5^ in 2016. Since its introduction in the Americas, more than 5.8 million Zika cases have been reported, as of December 2020,^6^ and more than 7452 cases were reported in 2020 alone.^7^ As of July 2019, 87 countries and territories reported cases of the ZIKV lineage that spread from French Polynesia.^8^ No vaccines exist for preventing ZIKV infections, thus prevention depends solely on the deployment of effective vector control measures and public health interventions.

Mathematical models can help to determine when and where future ZIKV outbreaks may occur and can retrospectively elucidate the effects of risk factors that facilitate spread. Here, we reviewed and compared models that have been adopted for investigating the geographic spread of ZIKV. We described their shared characteristics and evaluated the integration of various data sources for improving ZIKV surveillance. Finally, we discussed how studies have performed model validation to ensure robustness.

Box 1. Review search strategy

**Figure 1.**
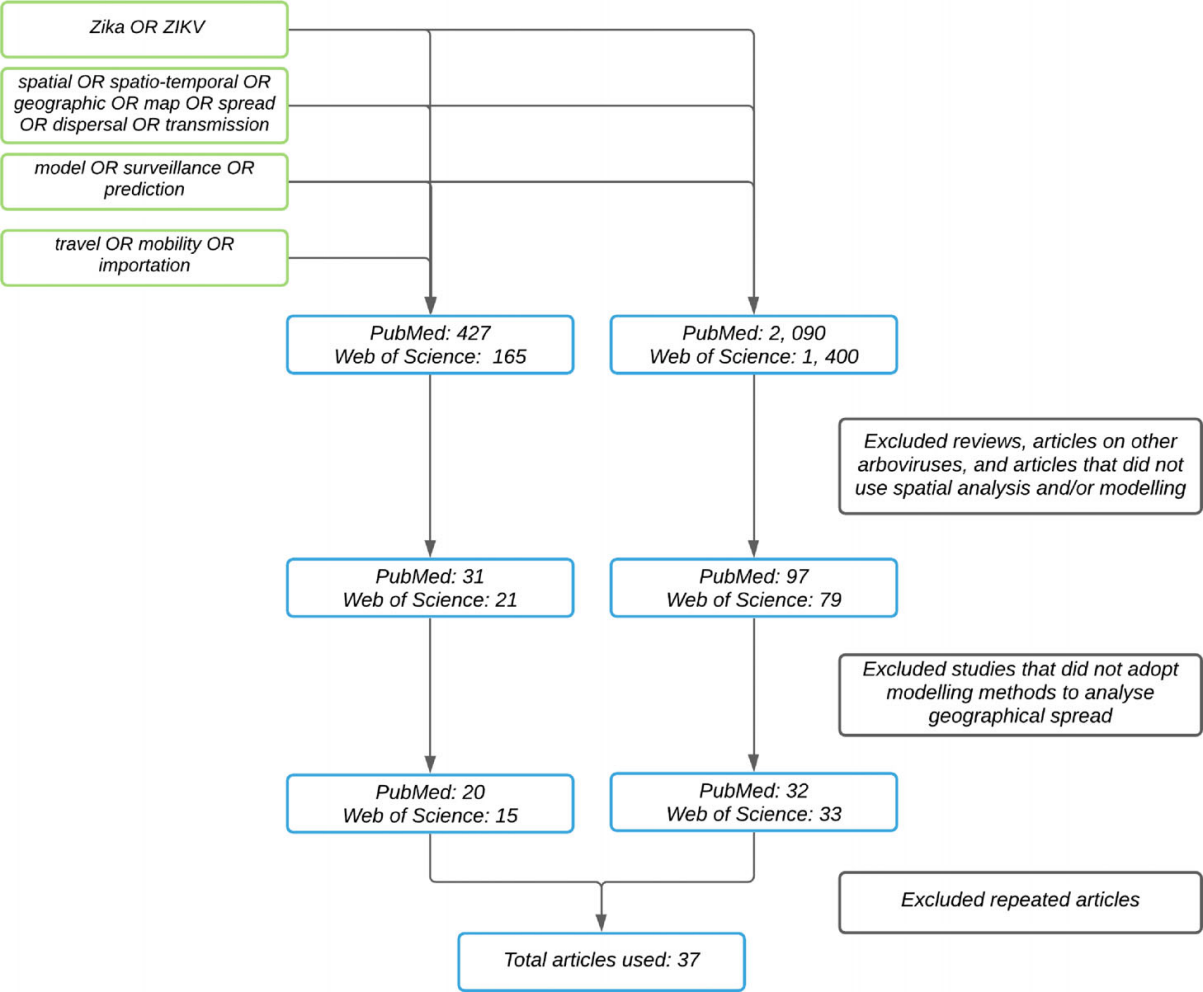
Flowchart illustrating the process of searching and retrieving the articles used in the review.

We conducted searches in PubMed and Web of Science for all published studies, as of 18 November 2020. We filtered studies using a combination of search strings (Figure 1) as follows:

- Zika OR ZIKV
- Spatial OR spatio-temporal OR geographic OR map OR spread OR dispersal OR transmission
- travel OR mobility OR importation
- model OR surveillance OR prediction

We then filtered studies using two exclusion criteria. First, we examined article abstracts and excluded articles that were (1) identified as reviews, (2) focused on other arboviruses and (3) did not use spatial analysis. Second, for the remaining articles, we evaluated the study content and excluded studies that did not adopt a modelling approach to look at the geographical spread of Zika. In total, we retained 37 studies that met our inclusion criteria.

## Characteristics of Zika modelling studies

**Figure 2.**
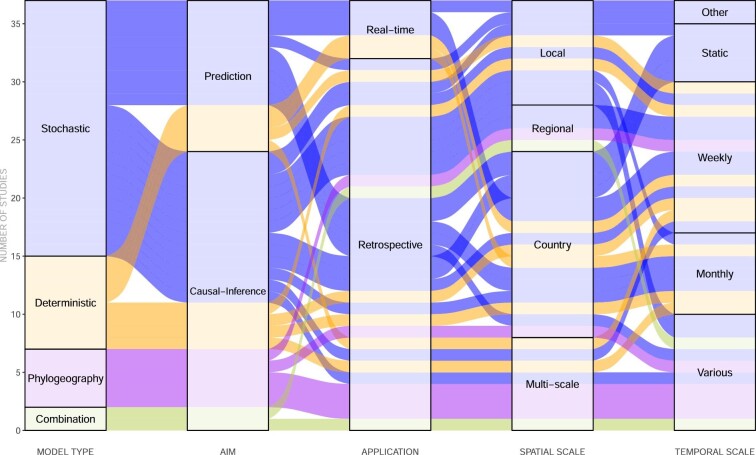
Characteristics of reviewed studies, summarised by model type, aim, application and scale.

**Figure 3.**
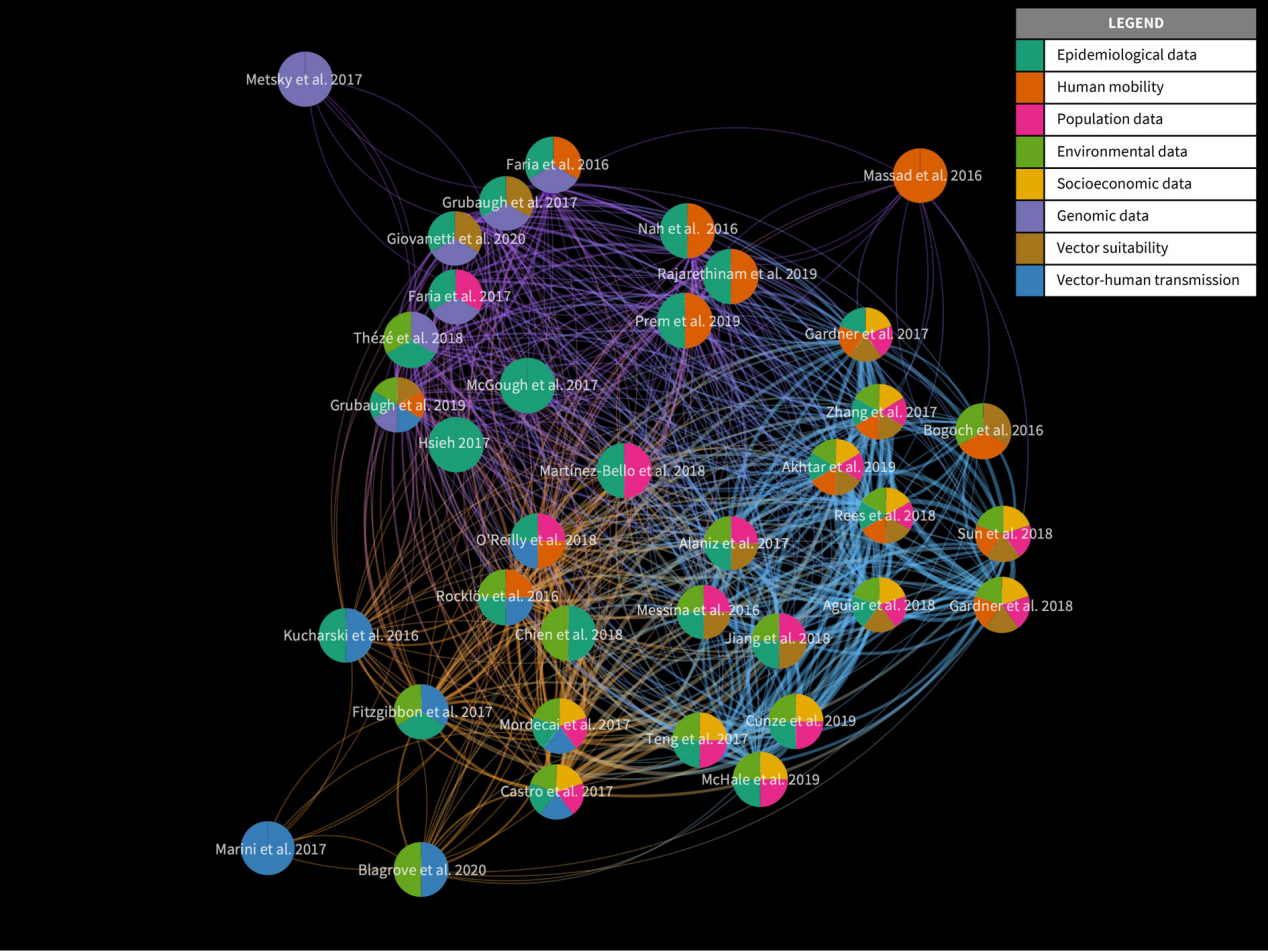
Network of reviewed studies, illustrating how studies are related to each other based on shared features. The pie chart at each node summarises the features incorporated in that particular study. Visualisation created using VOS viewer (v. 1.6.13).

We categorised studies by model type (stochastic or deterministic), aim, application, temporal and spatial scale. This is illustrated in Figure 2 as an alluvial diagram which shows how studies are connected by these categories. We compared model features and identified whether each model incorporated surveillance data and factors on human mobility, socioeconomic conditions, population, environmental conditions, mosquito suitability and mosquito-to-human transmission. We summarised this information using a network diagram (Figure 3), where each study is represented by a pie chart that illustrates the model features incorporated by that study. The thickness of each link represents the number of features shared between two studies and the colours of the links show how studies are clustered based on shared features.

## Model type

Stochastic, deterministic and phylogeographic methods have all been adopted to investigate ZIKV spread (Figure 2). Deterministic models use a set of input parameters, often from experimental findings or from the literature, to generate estimates on epidemiological characteristics that quantify spread,^9–13^ whereas stochastic models predict or infer retrospectively the process (or parameters) of spread using methods based on statistical theory.^14–24^ Deterministic models are effective for estimating mosquito–human interactions that facilitate infection^9–11^,^15,23,25,26^ but they are unable to account for the inherent stochastic nature of disease transmission.^10,12,13^ The majority of existing studies use stochastic models, which can flexibly integrate high-resolution information about environmental conditions and vector competence and thereby model fine-scale spatial heterogeneities in disease spread.^14,17,24,27^ However, stochastic models may rely heavily on the availability of sufficiently large and detailed surveillance data, which may not be available.^17,24,28^

Several studies have adopted phylogeographic methods, which have been increasingly adopted to rapidly assess the introduction and circulation of the viruses, including ZIKV. Phylogeographic modelling analyses can estimate the frequency and sources of ZIKV introduction into specific locations and new regions. Examples of this include studies of ZIKV in Latin America and the Caribbean^3,29,30^ and the USA.^31,32^ Some of these studies have adopted stochastic models in combination with phylogeography to predict ZIKV spread.^3,29,33^ However, it is worth noting that phylogeographic inferences may be sensitive to variation in sampling among locations.

## Model aim and application

We distinguished the modelling studies we reviewed according to two primary study objectives: causal-inference and prediction. The majority of studies are causal-inference (Figure 2), which aim to assess the contribution and interaction of factors in predicting ZIKV transmission.^22,26,34,35^ Additionally, they aim to identify the geographical origins of virus spread and epidemic history using phylogeographic methods.^3^,^30–32^ Predictive studies can either be stochastic or deterministic and may aim to estimate Zika incidence,^14,24,36^ importation of cases,^11,12,14^ distribution of risk^18,21^,^37–39^ and transmission potential.^15,23^ Both predictive and causal-inference models can be applied retrospectively to assess disease spread, which is the main application identified among the studies we reviewed. Predictive models can also be used to track disease spread with potential real-time applications.^13–15^,^20^

## Spatial and temporal scale

The geographic spread of ZIKV has been assessed extensively at the country and local level, with time intervals ranging from weekly to monthly (Figure 2). In addition to conducting analysis at the global level,^9,13,18,21,38,40^ studies have focused on the Latin American and Caribbean region,^20^,^28–30^,^33,36,41^ Africa and Asia-Pacific, Oceania, Europe^12,23^ and on specific countries such as the USA,^11,15,27,31^ Colombia,^16,19,34,42^ Brazil^3,10,37,43^ and Singapore.^22,44^ Multi-scale analysis^29,44^ and models that integrate data at different temporal and spatial scales^27,40,43^ can help to infer risk factors or make accurate predictions on geographic spread. This is especially useful for countries with passive surveillance systems that experience long delays in releasing official case data. However, aggregating data at different scales across a region with high spatial heterogeneity could result in inaccurate estimates of potential ZIKV risk.^16^

## ZIKV modelling objectives

Among the studies that we reviewed, we identified a set of common objectives that the authors aimed to achieve, which are elaborated below. Studies with similar objectives are also shown to be in the same clusters depicted in Figure 3.

## Mapping geographical distribution and risk

Prior to the availability of epidemiological data, mapping the environmental suitability of ZIKV can elucidate the potential distribution of exposure and risk of infection. This requires an understanding of the geographical distribution of *Aedes* sp. mosquito presence and the competence of vectors in transmitting ZIKV based on environmental factors, which can be inferred from mapping the mosquito's habitat suitability using ecological niche models.^21^ One of the earlier works by Messina et al.^21^ used ensemble boosted regression trees to map global environmental suitability for ZIKV. Combining this model output with disease occurrence, once it becomes available, can help to predict potential population exposure,^38^ transmission risk^41^ and risk factors^35^ at both regional and global scales.

In particular, integrating airline data and socioeconomic factors can help to characterise travel-related spread^39^ and the effects of socioeconomic factors on transmission.^37^ More recently, machine learning models, such as propagation neural network, gradient boosting machine and random forests, have also been applied to identify regions at risk globally.^18^ Similarly, spatio-temporal modelling using Bayesian inference can also be used to identify areas of high risk at the local level^19,42^ and the influence of travel history on risk.^44^

## Predicting local and imported infections

The frequency and risk of ZIKV infection, including local and imported infections, has been predicted using several methods. The rate of infection per population and region have been predicted using a stochastic spatial model,^24^ a dynamic neural network model^14^ and a deterministic model.^36^ A SEIR (susceptible, exposed, infectious, recovered) model framework has also been adopted to assess infection dynamics by quantifying vector–human interactions^25^ and the likelihood of sustaining mosquito-borne transmission.^15^ Furthermore, risk models developed by Gardner et al.^40^ that considered the vector competence of both *Aedes aegypti* and *Aedes Albopictus* have further revealed the risk of importation into new regions. Once the number of imported infections is known, the time and risk of importation from travellers can be used to understand the dynamics of local transmission^13,27^ and to dynamically model epidemic risk in real time.^15^ Exportation and importation risk profiles can also be generated to understand the risk of spread between geographical regions.^17^

The availability of near real-time data sources, such as Google searches, Twitter microblogs and ProMed (https://promedmail.org/), has also been leveraged to make timely predictions of ZIKV infections before official case data become available. Predictions of weekly ZIKV cases have been made 1–3 wk ahead of publication date in Latin American countries.^20^ The accuracy and geographic coverage of ‘big’ mobile and internet data are highly dependent on human–computer interactions and participation dynamics, thus reliability and biases from these sources may be limited.

## Quantifying transmission using R_0_

The basic reproduction number (R_0_) is a key metric used to define the capability of an infectious agent to proliferate^45^ and is commonly adopted in studies to understand transmission in populations. This number can vary across time (R_t_), as an estimate determined during an epidemic driven by human contact will differ as it transitions to an endemic state when the population has achieved advanced control efforts or herd immunity.

At the start of an epidemic, when public health interventions and population immunity are absent, R_0_ can be adopted as an effective metric to understand rapid spread. This is heavily dependent on the vector capacity of the mosquito, which is driven by temperature, the extrinsic incubation period of ZIKV, mosquito biting rate and vector abundance in a location.^15,23^ Given the optimal conditions for human–vector transmission, integrating data on local and regional travel can help assess transmission dynamics within and between populations^15,23,28^ and between human and vector to dissipate or sustain epidemics.^10^ Furthermore, by identifying and modelling *Aedes* sp. competence and transmission intensity under various temperature ranges, a range of R_0_ estimates by region can be derived to understand the distribution of transmission duration and risk during recent and future climate scenarios.^9,26^

## Reconstructing transmission pathways using phylogeography

Phylogeographic analyses can elucidate timely information on the rapid evolution and spatial spread of viral pathogens. Evolutionary trees combined with genetic and geolocation data have helped to determine the initial date of ZIKV circulation in the Americas, highlighting that local circulation began months before the first confirmed case.^32^ While it was costly and time-consuming to sequence large numbers of virus genomes in the past, more accessible and timely methods of pathogen genome sequencing are now available using portable genomic technologies. For example, nanopore instruments such as the MinION^46^ device can be rapidly and effectively adopted in remote and resource-low settings. For instance, ZIKV samples collected from the 2016 outbreaks in Brazil were generated within 48 h.^33^

Genomic data have been combined with epidemiological data to detect unreported outbreaks and to understand whether a virus is driven by importation or local transmission. By analysing viral genomes and epidemiological data, Faria et al.^33^ found that ZIKV was introduced to northeast Brazil as early as February 2014 prior to circulation in Brazil and detection in the Americas. Grubaugh et al.^29^ combined genomic data, passenger air travel information and local and travel-related infections to detect unreported outbreaks, such as one in Cuba that occurred a year after peak transmission in neighbouring islands. Applying genomic data to fill in gaps existing in epidemiological data has significant potential; however, given limited quantity and quality of travel data, combining travel surveillance in a joint framework remains a challenge that merits further exploration.

## Identifying risk factors and drivers of spread

Human mobility, climate change, urbanisation and socioeconomic disparities can drive variations in the geographic spread of ZIKV as well as outbreak intensity. Global human mobility, which has been increasing at unprecedented rates due to tourism, trade and migration, is a key determinant of global arbovirus distribution.^47^ The role of human mobility as a key predictor of ZIKV spread has been investigated by several studies, particularly when it concerns the risk of spread due to international travel^13,17,23,29,39^ and migration.^10,14,22,34,36^ Types of data and models used to quantify human mobility for Zika modelling are discussed further in the next section.

Climate factors, coupled with travel, can further drive changes in vector competence, particularly in locations with similar climate conditions that are conducive to *Aedes* habitat suitability. Temperature is a strong determinant of *A. aegypti* competence in transmitting Zika^48^ and has been incorporated in both deterministic and stochastic models to account for its role in driving Zika infections.^16,23,24,26,34,37,43^ Other factors such as precipitation and relative humidity also play an important role in fostering mosquito growth, which is conducive to Zika transmission.^16,18,21,34,39^

The role of socioeconomic factors in facilitating ZIKV spread remains inconclusive, as it varies by factor and spatial scale. While a strong negative association between gross domestic product and ZIKV transmission was found among countries^17,35^ this remains contested at the subnational level.^37^ Household conditions, namely, access to air conditioning, sanitation, piped water and garbage collection, were found to be linked with ZIKV spread and risk.^24,37^ Rees et al.^34^ found a negative relationship between poverty level and ZIKV detection for Colombia, but noted that this could be linked to under-reporting in poor areas due to limited healthcare access. Typically, urban settings have high landscape heterogeneity, providing optimal conditions for *A. aegypti* mosquitoes to thrive and foster arbovirus transmission to humans.^49,50^ Urbanisation has high predictive power for estimating ZIKV risk,^18,37^ along with population density, which is a significant risk factor.^17,35^

Variables relating to healthcare infrastructure have rarely been included as explanatory variables in existing ZIKV modelling studies. Gardner et al.^17^ used data on the number of hospital beds and number of physicians per 10 000 people to represent the distribution of healthcare infrastructure in their model. They found that these variables did not contribute significantly to country-level geographic spread and local transmission of ZIKV. Future studies should focus on incorporating indicators of healthcare access into their models to understand their role in predicting spread.

## Using human mobility to model ZIKV spread

### Quantifying mobility using travel data

Human mobility is a key driver of ZIKV spread and integrating this variable in spatial models can provide a valuable insight into spread within and across populations in near real time. Information on human movement has traditionally been collected from household travel surveys and censuses^51^; recently, such data have been acquired at higher resolutions from new digital sources. Table 1 summarises the strengths and weaknesses of existing human mobility indicators that have been used to track the spread of ZIKV and similar arboviruses. A more comprehensive list of data used to quantify human mobility for analysing disease and health risks has been reviewed elsewhere.^52^

**Table 1.** Indicators of human mobility for modelling arbovirus spread

Indicator of human mobility	Data source	Open access (Yes/No)	Spatial scale	Source
Passenger air travel	Global flights network (OpenFlights)	Yes	Global	13 ^*^
	International air transport association (IATA)	No	Global	39^*^;^17^^*^; 29^*^;^24^^*^; 40^*^; 14^*^;^23^^*^;^12^^*^;^3^^*^; 29^*^
	Official aviation guide (OAG)	No	Global	24 ^*^
Call data records	Operators and private companies	No	Local, regional	57;22^*^;^59^
Night-time lights brightness	Defense meteorological satellite program (DMSP)	Yes	Global coverage at 1 km x 1 km resolution	60
Global positioning system trackers	GPS data loggers used to track individual movement	No	Local (e.g. neighbourhood level)	61; 62; 63
	Google location history (GLH)	No		
	Cell phone towers	No		
Travel surveys	Government agencies	Yes	Local, regional	51
Infrastructure data	Road networks	Yes	Global	34^*^; 64
	Public transport contactless cards	No	Local	44 ^*^

*Studies that examined human mobility and its effects on ZIKV spread.

Data on passenger flights between locations can help to track ZIKV spread,^29,39,40^ but limit our understanding of movement to the origin and destination airports and rarely considers connections made between flights and travel made at the individual level.^53^ Call data records from mobile phones offer large coverage of populations and areas and can be leveraged to measure individual-level movement in near real time.^22,52^ Access to call data records is currently restricted to research groups and access is granted via a negotiated agreement with the operator, which makes their widespread use challenging. The release of personal individual details via mobile phone records to third parties is a privacy concern, thus censored via data aggregation and often provided as a small sample. Given these restrictions, ongoing research has outlined ways to improve accessibility,^54^ while initiatives such as the Open Algorithms project (https://www.opalproject.org) and FlowKit (https://flowkit.xyz) are working towards scaling the privacy-conscientious use of call data for research. Moreover, modelling efforts towards understanding the COVID-19 pandemic have further opened up these data sources to researchers.

### Quantifying mobility using mathematical models

When trip-level data are not available, human mobility can be quantified using models such as the gravity model of migration, a deterministic model that assumes that population movement between locations is proportional to some power of the population sizes of the origin and destination locations.^55^ O'Reilly et al.^36^ used a gravity model to assess the international spread of ZIKV in the Americas and found this model to be effective in fitting the data. However, assumptions of the gravity model, such as the lack of theoretical guidance for fitting empirical data, the requirement of existing traffic data to fit parameters and issues concerning modelling the flux in travel between two locations^56^ make it difficult to characterise complex travel behaviour across a large region.

To address these limitations, Simini et al.^56^ developed the radiation model, which is parameter-free and is based on a stochastic process that only requires information on population distribution. This model can be applied to estimate movement patterns in areas that lack mobility data and its predictions have generally aligned with observed mobility patterns, including long-term migration and population diffusion between areas. This method was widely adopted to estimate population movement between affected cities in Latin American countries during the 2015–2017 ZIKV outbreaks^36^ and similar arbovirus outbreaks.^57,58^ When information on population travel is limited, radiation models can effectively infer human mobility behaviour during an outbreak to estimate the magnitude of spread.

## Model validation

A good model for assessing disease spread is often validated for its accuracy, ensuring that the model, combined with assumptions, results in a sufficiently accurate representation of reality. Studies of ZIKV that aimed to forecast the number of infections or R_0_ at various spatial and temporal scales have validated their models by comparing the projected output with surveillance data not directly used to calibrate the model^15^,^22–24^ or by adopting statistical analysis to understand whether the predicted relationship was consistent with the data.^15,26,34^ While this is a simple way to validate model outputs, it may result in high bias if limited data were used. When the goal is to find the most suitable model among a selection of models, studies that modelled disease risk at the local and regional level have taken a criterion-based approach by comparing the Akaike Information Criterion (AIC)^16,19^ and the Deviance Information Criterion (DIC) when using Bayesian inference.^42,44^

When large amounts of data are available, k-fold cross-validation, a method for testing performance for machine learning models, and area-under-the-curve (AUC), a measure of predictive accuracy, can be adopted as quantitative diagnostic tools. Several studies that have explored ZIKV spread at regional and global levels have adopted k-fold cross-validation to train their models,^18,21,34,36,38^ particularly studies that have adopted an ecological niche modelling approach. Subsequently, AUC has been adopted to evaluate the predictive performance of these models.^18,21,34^ A subset of randomly selected data can be used as a test sample and the AUC can be applied to assess predictive accuracy^37^; when multiple models are present, AUCs can be compared.^13^ AUC can also be combined with the receiving operating characteristic curve analysis to further examine a model's predictive ability.^14,35^

### Conclusions

Mathematical models can enhance surveillance and help identify potential risk factors that drive ZIKV spread. We reviewed studies that investigated the geographical spread of ZIKV, discussed common model features and examined the role of various transmission risk factors. We highlighted the value of adopting novel data sources that characterise human mobility to monitor transmission in real time, as well as the potential integration of such data with environmental and socioeconomic data phylogeographic methods. We also summarised model validation strategies and recommend their implementation. Our review provides an overview of model characteristics that future studies should consider when modelling the geographic spread of ZIKV and other arboviruses to prevent future outbreaks.

## Data Availability

All search filters used in this review are stated in the text.
